# Oral Factors Associated with Swallowing Function in Independent Elders

**DOI:** 10.3290/j.ohpd.a45071

**Published:** 2020-09-04

**Authors:** Yoshihiro Shimazaki, Mizuki Saito, Toshiya Nonoyama, Yasushi Tadokoro

**Affiliations:** a Professor, Department of Preventive Dentistry and Dental Public Health, School of Dentistry, Aichi Gakuin University, Nagoya, Japan. Idea, hypothesis, experimental design, data analysis, wrote the manuscript.; b Postgraduate Student, Department of Preventive Dentistry and Dental Public Health, School of Dentistry, Aichi Gakuin University, Nagoya, Japan. Experimental design, data analysis, wrote the manuscript, contributed substantially to discussion.; c Assistant Professor, Department of Preventive Dentistry and Dental Public Health, School of Dentistry, Aichi Gakuin University, Nagoya, Japan. Data analysis, proofread the manuscript, contributed substantially to discussion.; d Chairman, Mie Dental Association, Tsu, Japan. Contributed to the paper: idea, data collection, proofread the manuscript, contributed substantially to discussion.

**Keywords:** swallowing function, tooth, dentures, xerostomia

## Abstract

**Purpose:**

This study clarified the oral factors related to swallowing function using the results of dental check-ups among community-dwelling independent elders.

**Materials and Methods:**

Data on oral and medical health check-ups from 4,676 participants aged 75 and 80 years were analysed. Swallowing function was evaluated using the repetitive saliva swallowing test (RSST), which assesses the ability to swallow saliva. Swallowing three or more times during a 30 s period was recorded as normal, while swallowing twice or less in 30 s was considered to indicate swallowing difficulty. Multivariate logistic regression analyses were performed to examine the relationship between oral factors and swallowing function.

**Results:**

In all participants, the odds ratio (OR) for swallowing difficulty was 3.42 (95% confidence interval (CI): 1.78–6.55) and 6.68 (95% CI: 1.97–22.64) among those who had 10–19 teeth without dentures and those who had 0–9 teeth without dentures, respectively, compared to individuals with ≥ 20 teeth without dentures. Those with moderate or severe dry mouth had a statistically significantly higher OR (8.01, 95% CI: 4.84–13.27) for swallowing difficulty than those without dry mouth. Among dentate participants, in addition to statistically significant variables in the analysis among all participants, those with abundant dental plaque showed a significantly higher OR (2.58, 95% CI: 1.54–4.32) for swallowing difficulty compared to those with no or slight dental plaque.

**Conclusion:**

These results suggest that oral factors such as having few teeth without dentures, dry mouth, and poor oral hygiene are related to swallowing function in elders.

The ageing population in Japan is increasing rapidly. Indeed, the proportion of elders aged 65 years or over in the total population was 27.7% in 2017, and is predicted to reach approximately 38% in 2060.^10^

With the increase in the proportion of older individuals, disease structures have changed, and pneumonia was the third largest contributor to all-cause death following cancer and heart disease from 2011 to 2016 in Japan.^9^ Among pneumonia cases occurring in elders, aspiration pneumonia due to decreased swallowing function is frequently observed. Swallowing function is affected by ageing, and it has been shown that elders show decreased swallowing function compared to young people.^19,31^ Specifically, many elders who require nursing care have decreased swallowing function.^19^ It has been shown that the onset of pneumonia in elders requiring nursing care is greatly affected by swallowing function.^25^ Therefore, prevention of swallowing function deterioration in elders is important to prevent the onset of pneumonia and reduce the risk of death from pneumonia.

Many elders have lost a large number of teeth, and a decreased number of teeth is associated with reduced swallowing function.^4,17^ Those who have lost many teeth frequently use dentures to recover chewing function. However, if those who are edentulous do not use dentures, movements related to swallowing function in the oral cavity are affected.^15,28^ Previous studies have suggested that oral factors such as the number of teeth and prosthetic condition are closely related to swallowing function.^4,15,17,28^ However, there is little information concerning how the correlation between number of teeth and the prosthetic situation is related to the deterioration of swallowing function in independent elders.

It is important to clarify the relationship between number of teeth, the use of dental prostheses, and other oral cavity factors with deterioration of swallowing function in elders to promote oral and systemic healthcare measures. In this study, we clarified the oral factors associated with swallowing function using the results of dental check-ups among community-dwelling independent elders over 75 years of age.

## Materials and Methods

### Study Population

The participants of this study were insured by the public healthcare insurance system for individuals aged ≥ 75 years in Japan. Since 2014, the government has subsidised oral health examinations for elders under each prefectural insurer, called ‘wide area unions for the late-stage medical care system’, and the Mie Prefecture insurer covers oral health examinations for those aged 75 and 80 years. This cross-sectional study was performed using existing records obtained from oral health examinations covered by the insurer of elders aged ≥ 75 years in Mie Prefecture.

In September 2014, the insurer sent an oral health examination ticket to 75 and 80-year-old individuals residing in Mie Prefecture. Oral examinations were performed at a registered Mie Dental Association Clinic from 1 October to 30 November 2014. Oral and medical health examination data from 4676 participants aged 75 or 80 years were analysed, after excluding 308 people with missing data. As the health examinations were conducted as part of oral health check-ups and were not specifically conducted for this study, consent was not obtained from the participants. We obtained permission from the insurer to use the anonymised data for this study. This study was approved by the Institutional Review Board (IRB) of Aichi Gakuin University School of Dentistry (approval number 443), and was conducted in full accordance with the World Medical Association Declaration of Helsinki. This study conforms to the STROBE guidelines for human observational studies.

### Oral Health Examination

Dentists at each dental clinic conducted oral health examinations using detailed manuals to standardise the assessment of oral health status. The condition of each tooth in each participant was recorded as sound, decayed, filled, or missing. The number of remaining teeth was calculated as the total number of sound, decayed, and filled teeth after excluding third molars. The community periodontal index (CPI) was used to assess periodontal status^29^ because this method is commonly used in public oral health examinations in Japan. The oral cavity of each participant was divided into six sextants. The index tooth numbers were 11, 16, 17, 26, 27, 31, 36, 37, 46, and 47. Measurements were made at six sites (mesiobuccal, mid-buccal, distobuccal, distolingual, mid-lingual, and mesiolingual) in each index tooth. The CPI scores were coded as follows: healthy (code 0); bleeding after probing (code 1); dental calculus detected by probing (code 2); 4–5 mm shallow pocket (code 3); ≥ 6 mm deep pocket (code 4); and relevant tooth missing (code X). To assess oral hygiene status, the presence of dental plaque was evaluated from all remaining teeth using the plaque index^23^ and was coded as follows: 0–1, no or slight plaque; 2, moderate plaque; and 3, abundant plaque. A clinical diagnosis of dry mouth was made using a classification scale that included the condition of the oral mucosa, and was coded as follows: grade 0, non-dry (does not show condition 1–3); grade 1, saliva shows viscosity; grade 2, saliva shows tiny bubbles on the tongue; and grade 3, dry tongue without viscosity and little or no saliva present.^6^

### Swallowing Function

Swallowing function was evaluated using the repetitive saliva swallowing test (RSST), which assesses the potential to swallow saliva.^5,11^ The first and second finger pads were placed on the hyoid bone and the laryngeal prominence. The participants were asked to swallow their saliva repeatedly. A dentist confirmed laryngeal elevation with the swallowing reflex and counted the frequency of voluntary swallows within 30 s. Swallowing three or more times in 30 s was recorded as normal, while swallowing twice or less in 30 s was considered to indicate swallowing difficulty.^12,13^

### Questionnaire

Information concerning the participants’ current medical history (diabetes, stroke, cardiac disease, cancer, lung disorders, or osteoporosis), smoking habit (current or never/past) and body mass index (BMI; kg/m^2^) was obtained using self-administered questionnaires. The use of dentures was defined by whether the participants used dentures routinely or had dentures but did not use them routinely.

### Statistical Analysis

Data from 4676 participants were included in the analyses. A combination variable of the number of teeth and use of dentures was calculated as follows: (number of teeth/use of denture) ≥ 20 teeth/not in use; ≥ 20 teeth/in use; 10–19 teeth/not in use; 10–19 teeth/in use; 0–9 teeth/not in use; and 0–9 teeth/in use. In analyses of the 4406 dentate participants, a variable combining the number of teeth and use of dentures was calculated as follows: (number of teeth/use of denture) ≥ 20 teeth/not in use; ≥ 20 teeth/in use; 10–19 teeth/not in use; 10–19 teeth/in use; 1–9 teeth/not in use; and 1–9 teeth/in use. The presence of decayed teeth was divided into two categories (0 or ≥ 1). The participants were divided into three categories based on the CPI (codes 0–2, 3–4, or X), where code X is a dummy code corresponding to the absence of representative teeth. Dry mouth was divided into three grades as follows: 0, 1, or 2–3. BMI was divided into the following three categories: <20 (underweight); 20–24.9 (normal weight); and ≥ 25 (overweight).

Differences in the proportion of categorical variables were evaluated using Pearson’s chi-square test. We performed logistic regression analyses to examine the associations of age, sex, number of teeth, use of dentures, the combination variable of the number of teeth and use of dentures, dry mouth, smoking habit, BMI, medical history, presence of decayed teeth, CPI, and dental plaque with swallowing function. Both unadjusted and covariate-adjusted odds ratios (ORs) and confidence intervals (CIs) were calculated for swallowing difficulty (RSST ≤ 2). The presence of decayed teeth, CPI, and dental plaque were used only in the analysis among dentate participants. The variables of the number of teeth and use of dentures were subjected to multivariate analysis separately from the combination variable of the number of teeth and use of dentures. The variables of the number of teeth and use of dentures, and other variables that were statistically significant in the bivariate analysis, were included in the multivariate logistic regression analysis. All statistical analyses were performed using SPSS ver. 24.0 (SPSS Inc.). A p value < 0.05 was considered to indicate statistical significance.

## Results

In 2014, there were 17,338 individuals aged 75 years and 16,040 individuals aged 80 years in Mie Prefecture. A total of 2865 (16.5%) and 2119 (13.2%) 75- and 80-year-old individuals underwent oral health examinations, respectively. We analysed the data of 4676 individuals who met the requirements for analysis, including 2701 participants aged 75 years and 1975 participants aged 80 years.

Tables 1 and 2 shows the characteristics of the study participants according to swallowing function. There were 181 (3.9%) individuals with swallowing difficulty overall and 163 (3.7%) among the dentate participants. Age, sex, number of teeth, the combination variable of number of teeth and denture use, dry mouth, BMI, diabetes, stroke, and osteoporosis were statistically significantly associated with swallowing function among all participants (Table 1). Among dentate participants, age, sex, number of teeth, the combination variable of number of teeth and denture use, dry mouth, BMI, diabetes, stroke, osteoporosis, presence of decayed teeth, and the presence of dental plaque were statistically significantly associated with swallowing function (Table 2).

**Table 1:** Characteristics of participants according to swallowing function in all participants (N = 4676)

Variable	Swallowing function (RSST)		Dependent variable: RSST (≥ 3 = 0, ≤ 2 = 1)	
	Normal (≥ 3)	Difficulty (≤ 2)	Crude OR (95% CI)	p value
All participants	(N = 4495)	(N = 181)		
	N (%)			
**Age**				
75 years	2624 (58.4)	77 (42.5)	1	
80 years	1871 (41.6)	104 (57.5)	1.89 (1.40–2.56)	< 0.001
Sex				
Male	2066 (46.0)	55 (30.4)	1	
Female	2429 (54.0)	126 (69.6)	1.95 (1.41–2.69)	< 0.001
**Number of teeth**				
≥ 20	2483 (55.2)	70 (38.7)	1	
10–19	1085 (24.1)	60 (33.1)	1.96 (1.38–2.79)	< 0.001
0–9	927 (20.6)	51 (28.1)	1.95 (1.35–2.82)	< 0.001
**Use of dentures**				
Not in use	1981 (44.1)	71 (39.2)	1	
In use	2514 (55.9)	110 (60.8)	1.22 (0.90–1.66)	0.20
**Teeth/Denture**				
≥ 20/not in use	1852 (41.2)	53 (29.3)	1	
≥ 20/in use	631 (14.0)	17 (9.4)	0.94 (0.54–1.64)	0.83
10–19/not in use	114 (2.5)	14 (7.7)	4.29 (2.31–9.97)	< 0.001
10–19/in use	971 (21.6)	46 (25.4)	1.66 (1.11–2.48)	< 0.05
0–9/not in use	15 (0.3)	4 (2.2)	9.32 (2.99–29.03)	< 0.001
0–9/in use	912 (20.3)	47 (26.0)	1.80 (1.21–2.69)	< 0.01
**Dry mouth**				
Grade 0	3494 (77.7)	78 (43.1)	1	
Grade 1	886 (19.7)	78 (43.1)	3.94 (2.86–5.44)	< 0.001
Grade 2–3	115 (2.6)	25 (13.8)	9.74 (5.98–15.85)	< 0.001
**Smoking habit**				
Never/past	4272 (96.0)	171 (95.5)	1	
Current	180 (4.0)	8 (4.5)	1.11 (0.54–2.29)	0.78
**BMI (kg/m^2^)**				
<20	959 (21.3)	54 (29.8)	1.73 (1.23–2.15)	< 0.01
20–24.9	2736 (60.9)	89 (49.2)	1	
≥ 25	800 (17.8)	38 (21.0)	1.46 (0.99–5.27)	0.06
**Diabetes**				
No	4012 (89.3)	151 (83.4)	1	
Yes	483 (10.7)	30 (16.6)	1.65 (1.10–2.47)	< 0.05
**Stroke**				
No	4405 (98.0)	171 (94.5)	1	
Yes	90 (2.0)	10 (5.5)	2.86 (1.46–5.60)	< 0.01
**Cardiac disease**				
No	3911 (87.0)	158 (87.3)	1	
Yes	584 (13.0)	23 (12.7)	0.98 (0.62–1.52)	0.91
**Cancer**				
No	4249 (94.5)	175 (96.7)	1	
Yes	246 (5.5)	6 (3.3)	0.59 (0.26–1.35)	0.21
**Lung disorder**				
No	4387 (97.6)	174 (96.1)	1	
Yes	108 (2.4)	7 (3.9)	1.63 (0.75–3.56)	0.22
**Osteoporosis**				
No	3973 (88.4)	149 (82.3)	1	
Yes	522 (11.6)	32 (17.7)	1.64 (1.10–2.42)	< 0.05

N, number of participants; RSST, repetitive saliva swallowing test; OR, odds ratio; CI, confidence interval; BMI, body mass index.

**Table 2:** Characteristics of participants according to swallowing function among dentate participants (N = 4406)

Variable	Swallowing function (RSST)		Dependent variable: RSST (≥ 3 = 0, ≤ 2 = 1)	
	Normal (≥ 3)	Difficulty (≤ 2)	Crude OR (95% CI)	p value
Dentate participants	(N = 4243)	(N = 163)		
	N (%)			
**Age**				
75 years	2531 (59.7)	73 (44.8)	1	
80 years	1712 (40.3)	90 (55.2)	1.82 (1.33–2.50)	< 0.001
**Sex**				
Male	1952 (46.0)	46 (28.2)	1	
Female	2291 (54.0)	117 (71.8)	2.17 (1.53–3.06)	< 0.001
**Number of teeth**				
≥ 20	2480 (58.4)	70 (42.9)	1	
10–19	1082 (25.5)	60 (36.8)	1.96 (1.38–2.79)	< 0.001
1–9	681 (16.0)	33 (20.2)	1.72 (1.13–2.62)	< 0.05
**Use of dentures**				
Not in use	1978 (46.6)	70 (42.9)	1	
In use	2265 (53.4)	93 (57.1)	1.16 (0.85–1.59)	0.36
**Teeth/Denture**				
≥ 20/not in use	1851 (43.6)	53 (32.5)	1	
≥ 20/in use	629 (14.8)	17 (10.4)	0.94 (0.54–1.64)	0.84
10–19/not in use	114 (2.7)	14 (8.6)	4.29 (2.31–7.96)	< 0.001
10–19/in use	968 (22.8)	46 (28.2)	1.66 (1.11–2.48)	< 0.05
1–9/not in use	13 (0.3)	3 (1.8)	8.06 (2.23–29.13)	< 0.01
1–9/in use	668 (15.7)	30 (18.4)	1.57 (0.99–2.48)	0.05
**Dry mouth**				
Grade 0	3321 (78.3)	72 (44.2)	1	
Grade 1	817 (19.3)	71 (43.6)	4.01 (2.86–5.61)	< 0.001
Grade 2–3	105 (2.5)	20 (12.3)	8.79 (5.16–14.96)	< 0.001
**Smoking habit**				
Never/past	4039 (96.1)	155 (96.3)	1	
Current	163 (3.9)	6 (3.7)	0.96 (0.42–2.20)	0.92
**BMI (kg/m^2^)**				
<20	897 (21.1)	50 (30.7)	1.85 (1.29–2.66)	< 0.001
20–24.9	2589 (61.0)	78 (47.9)	1	
≥ 25	757 (17.8)	35 (21.5)	1.54 (1.02–2.31)	< 0.05
**Diabetes**				
No	3795 (89.4)	134 (82.2)	1	
Yes	448 (10.6)	29 (17.8)	1.83 (1.21–2.77)	< 0.01
**Stroke**				
No	4160 (98.0)	153 (93.9)	1	
Yes	83 (2.0)	10 (6.1)	3.28 (1.67–6.44)	< 0.001
**Cardiac disease**				
No	3693 (87.0)	142 (87.1)	1	
Yes	550 (13.0)	21 (12.9)	0.99 (0.62–1.58)	0.98
**Cancer**				
No	4012 (94.6)	160 (98.2)	1	
Yes	231 (5.4)	3 (1.8)	0.33 (0.10–1.03)	0.06
**Lung disorder**				
No	4144 (97.7)	157 (96.3)	1	
Yes	99 (2.3)	6 (3.7)	1.60 (0.69–3.70)	0.27
**Osteoporosis**				
No	3744 (88.2)	135 (82.8)	1	
Yes	499 (11.8)	28 (17.2)	1.56 (1.03–2.36)	< 0.05
**Decayed teeth**				
0	2715 (64.0)	86 (52.8)	1	
≥ 1	1528 (36.0)	77 (47.2)	1.59 (1.16–2.18)	< 0.01
**CPI**				
Code 0–2	1592 (37.5)	51 (31.3)	1	
Code 3–4	2465 (58.1)	101 (62.0)	1.28 (0.91–1.80)	0.16
Code X	186 (4.4)	11 (6.7)	1.85 (0.95–3.60)	0.07
**Dental plaque**				
No or slight	1795 (42.3)	48 (29.4)	1	
Moderate	2169 (51.1)	85 (52.1)	1.47 (1.02–2.10)	< 0.05
Abundant	279 (6.6)	30 (18.4)	4.02 (2.51–6.46)	< 0.001

N, number of participants; RSST, repetitive saliva swallowing test; OR, odds ratio; CI, confidence interval; BMI, body mass index; CPI, Community Periodontal Index.

Table 3 shows the relationship between swallowing function and the dependent variables, including oral factors, in a multivariate logistic regression analysis. Having less than 20 teeth and the presence of dry mouth had a statistically significantly higher OR for swallowing difficulty, and use of dentures had a statistically significantly lower OR for swallowing difficulty. As to the combination variable of the number of teeth and use of dentures, compared to those with 20 or more teeth without dentures, the OR for swallowing difficulty of the participants with 10–19 teeth without dentures and of those with 0–9 teeth without dentures was 3.42 (95% CI: 1.78–6.55) and 6.68 (95% CI: 1.97–22.64), respectively. Those with dry mouth of grade 2–3 had a statistically significantly higher OR (8.01, 95% CI: 4.84–13.27) for swallowing difficulty, compared to individuals without dry mouth.

**Table 3:** Relationship between swallowing function and independent variables, including oral factors, in multivariate logistic regression analysis in all participants (N = 4676)

Variable	Dependent variable: RSST (≥ 3 = 0, ≤ 2 = 1)			
	Model 1		Model 2	
	Adjusted OR (95% CI)	p value	Adjusted OR (95% CI)	p value
**Age**				
75 years	1		1	
80 years	1.62 (1.18–2.21)	< 0.01	1.61 (1.17–2.20)	< 0.01
**Sex**				
Male	1		1	
Female	1.71 (1.21–2.41)	< 0.01	1.73 (1.22–2.44)	< 0.01
**Number of teeth**				
≥ 20	1		–	
10–19	2.26 (1.41–3.62)	< 0.001	–	
0–9	2.21 (1.30–3.75)	< 0.01	–	
**Use of dentures**				
Not in use	1		–	
In use	0.59 (0.37–0.93)	< 0.05	–	
**Teeth/Denture**				
≥ 20/not in use	–		1	
≥ 20/in use	–		0.96 (0.55–1.69)	0.90
10–19/not in use	–		3.42 (1.78–6.55)	< 0.001
10–19/in use	–		1.37 (0.90–2.07)	0.14
0–9/not in use	–		6.68 (1.97–22.64)	< 0.01
0–9/in use	–		1.38 (0.91–2.09)	0.13
**Dry mouth**				
Grade 0	1		1	
Grade 1	3.51 (2.53–4.87)	< 0.001	3.52 (2.54–4.89)	< 0.001
Grade 2–3	8.15 (4.94–13.45)	< 0.001	8.01 (4.84–13.27)	< 0.001
**BMI (kg/m^2^)**				
< 20	1.41 (0.98–2.02)	0.06	1.41 (0.98–2.02)	0.06
20–24.9	1		1	
≥ 25	1.27 (0.85–1.89)	0.25	1.28 (0.86–1.91)	0.23
**Diabetes**				
No	1		1	
Yes	1.93 (1.26–2.94)	< 0.01	1.91 (1.25–2.91)	< 0.01
**Stroke**				
No	1		1	
Yes	2.91 (1.43–5.92)	< 0.01	3.01 (1.48–6.14)	< 0.01
**Osteoporosis**				
No	1		1	
Yes	1.35 (0.89–2.04)	0.16	1.36 (0.90–2.07)	0.15

N, number of participants; RSST, repetitive saliva swallowing test; OR, odds ratio; CI, confidence interval; BMI, body mass index.

Table 4 shows the relationship between swallowing function and dependent variables, including oral factors, in a multivariate logistic regression analysis among dentate participants. In addition to the statistically significant variables in the analysis among all participants, being underweight (BMI <20 kg/m^2^) had a statistically significantly higher OR (1.49, 95% CI: 1.02–2.18) for swallowing difficulty compared to being of a normal weight (BMI 20–24.9) and abundant dental plaque accumulation had a statistically significantly higher OR (2.58, 95% CI: 1.54–4.32) for swallowing difficulty compared to those with no or slight dental plaque.

**Table 4:** Relationship between swallowing function and independent variables, including oral factors, in multivariate logistic regression analysis among dentate participants (N = 4406)

Variable	Dependent variable: RSST (≥ 3 = 0, ≤ 2 = 1)			
	Model 1		Model 2	
	Adjusted OR (95% CI)	p value	Adjusted OR (95% CI)	p value
**Age**				
75 years	1		1	
80 years	1.49 (1.07–2.07)	< 0.05	1.47 (1.06–2.05)	< 0.05
**Sex**				
Male	1		1	
Female	2.01 (1.39–2.92)	< 0.001	2.04 (1.40–2.97)	< 0.001
**Number of teeth**				
≥ 20	1		–	
10–19	2.06 (1.28–3.32)	< 0.01	–	
1–9	1.91 (1.08–3.38)	< 0.05	–	
**Use of dentures**				
Not in use	1		–	
In use	0.67 (0.42–1.07)	0.09	–	
**Teeth/Denture**				
≥ 20/not in use	–		1	
≥ 20/in use	–		1.04 (0.59–1.84)	0.88
10–19/not in use	–		3.13 (1.62–6.06)	< 0.001
10–19/in use	–		1.41 (0.93–2.14)	0.11
1–9/not in use	–		5.99 (1.49–24.14)	< 0.05
1–9/in use	–		1.33 (0.82–2.13)	0.25
**Dry mouth**				
Grade 0	1		1	
Grade 1	3.42 (2.42–4.84)	< 0.001	3.45 (2.44–4.88)	< 0.001
Grade 2–3	6.50 (3.72–11.37)	< 0.001	6.43 (3.66–11.29)	< 0.001
**BMI (kg/m^2^)**				
<20	1.50 (1.03–2.19)	< 0.05	1.49 (1.02–2.18)	< 0.05
20–24.9	1		1	
≥ 25	1.25 (0.82–1.91)	0.30	1.26 (0.83–1.93)	0.28
**Diabetes**				
No	1		1	
Yes	2.10 (1.36–3.25)	< 0.01	2.08 (1.34–3.22)	< 0.01
**Stroke**				
No	1		1	
Yes	2.99 (1.43–6.24)	< 0.01	3.10 (1.83–6.49)	< 0.01
**Osteoporosis**				
No	1		1	
Yes	1.30 (0.83–2.02)	0.25	1.30 (0.83–2.02)	0.25
**Decayed teeth**				
0	1		1	
≥ 1	1.37 (0.98–1.92)	0.06	1.31 (0.84–2.05)	0.23
**Dental plaque**				
No or slight	1		1	
Moderate	1.11 (0.76–1.61)	0.60	1.10 (0.76–1.61)	0.62
Abundant	2.56 (1.53–4.27)	< 0.001	2.58 (1.54–4.32)	< 0.001

N, number of participants; RSST, repetitive saliva swallowing test; OR, odds ratio; CI, confidence interval; BMI, body mass index.

## Discussion

In this study, we examined oral factors related to decreased swallowing function, using data obtained from dental examinations for independent elders who live in the community.

Analysis of the relationship of the combination variable of number of teeth and use of dentures with swallowing function revealed that a decrease in swallowing function was observed in those who had few teeth and did not use dentures. A previous cross-sectional study showed that men with many teeth had a statistically significantly lower OR for dysphagia.^4^ A 5-year cohort study targeting community-dwelling independent elders showed that those with few teeth were at a high risk of developing a swallowing disorder.^17^ Loss of numerous teeth is thought to increase the risk of swallowing function deterioration. In elders who require nursing care, the risk of dysphagia was high in those who did not have molar occlusal sites, including with prosthesis use, such as dentures and implants, or who did not have mandibular stability due to the lack of posterior support for occlusion with natural teeth or dental prostheses.^15,28^ Besides decreasing the number of teeth, occlusion of molar teeth, including prosthesis use, plays an important role in swallowing function. When edentulous individuals do not wear dentures, it has been shown that the occurrence of laryngeal penetration was statistically significantly higher compared with dentate subjects. However, there was no difference in laryngeal invasion between edentulous subjects wearing dentures and dentate subjects.^32^ Moreover, a lower number of teeth and cognitive impairment directly affected whether an individual wore their dentures, and both having many teeth and wearing dentures were positively associated with good swallowing function evaluated using cervical auscultation.^2^ Prevention of tooth loss is an important factor for suppressing the deterioration of swallowing function. Even if individuals lose many teeth, they may be able to suppress swallowing function deterioration by using prosthetic appliances.

Several reports have described the mechanisms underlying the effect of a decreased number of teeth and unused dentures on swallowing function decline. It is suggested that if edentulous subjects do not use dentures, bolus transport during eating is affected, which may increase the risk of dysphagia.^30^ In a previous study, the movement range of the mandible, hyoid bone, and larynx was large when dentures were not worn, which may compensate for oropharyngeal movement spatial memory to avoid temporal changes in oropharyngeal swallowing.^18^ When edentulous elders did not use dentures, tongue-lip motion was hyperactive, which may affect swallowing function.^33^ Moreover, it is thought that the absence of dentures among edentulous subjects leads to the deterioration of swallowing function due to decreased tongue-palate contact.^7^ However, in a previous study, the deglutition time was shorter when edentulous subjects wore dentures.^3^ As the number of teeth decreases, the occlusal area of the molar section decreases and the mandibular jaw position becomes unstable, which affects swallowing motion. The use of prosthetic appliances is thought to affect not only occlusion and chewing but also movements related to maintaining the stability of the mandibular jaw position. Furthermore, it exerts smoothing effects on swallowing motion and suppresses deterioration of swallowing function.

In this study, dry mouth was strongly associated with decreased swallowing function. A previous study showed that individuals aware of xerostomia had a statistically significantly higher OR for dysphagia.^4^ In independent elderly individuals, a subjective feeling of dry mouth was associated with a decline in swallowing function, independently of a decreased number of teeth.^16^ Dry mouth is reportedly more common in women than in men,^22^ and the risk of swallowing difficulty was higher in women in this study. Therefore, dry mouth may be related to the higher risk of swallowing difficulty in women. In contrast, both sex and dry mouth were statistically significantly related to swallowing difficulty in the multivariate analysis, so it is considered that each of these factors affects swallowing function independently. Saliva plays a variety of roles in the oral cavity, including contributing to taste, mastication, digestion, and swallowing.^20^ Mouth dryness is largely influenced by saliva secretion; thus, if it is decreased, movements associated with swallowing become difficult, resulting in swallowing function decline. Indeed, swallowing problems were reported in a previous study that investigated swallowing function in elderly individuals with low salivary secretion.^21^ Reductions in saliva secretion can result in dental plaque deposits due to a decreased ability of the oral cavity to self-clean. Although there was a statistically significant association between plaque accumulation and swallowing function in this study, it is conceivable that the observed association indicates that the plaque accumulation was caused by decreased oral motor dysfunction.^24^ Significant dental plaque can increase the risk of pneumonia due to aspiration.^1^ It may be necessary to implement prevention measures to combat oral drying in those experiencing xerostomia, including the use of moisturising agents, to reduce the risk of pneumonia following a decline in swallowing function.

Swallowing difficulty was observed in 4% of the relatively healthy community-dwelling independent elderly individuals in this study, and underweight elders tended to have swallowing difficulty. Therefore, malnutrition in elders may be linked to decreased swallowing function. Also, the proportion of elders with swallowing difficulty tends to be higher in those requiring a high level of care.^15^ A previous cross-sectional study reported a statistically significant relationship between decreased swallowing function and malnutrition in elderly people who needed nursing care.^26^ Another longitudinal study showed that elderly individuals with a swallowing disorder at baseline had significantly higher risk of malnutrition at follow-up.^14^ Swallowing dysfunction also increased the risk of a decline in the ability to perform activities of daily living (ADLs).^27^ Decreased swallowing function may lead to the development of frailty in the elderly. Because swallowing function and nutritional status are closely related, preventing a decrease in swallowing function and ensuring adequate nutrition would facilitate prevention of the decline in ADLs in elders.

In this study, the RSST was used as an evaluation method of swallowing function. To evaluate and diagnose clinical dysphagia, videofluorography (VF) studies are used.^8^ Although the accuracy of the RSST is inferior to that of VF, the sensitivity and specificity of the RSST were 0.98 and 0.66, respectively, when it was used as a screening test for dysphagia.^12^ In addition, the RSST is an effective screening tool to predict oral intake in elderly patients with acute pneumonia.^11^ Because the RSST makes use of a simple method that can evaluate deterioration of swallowing function without the use of special equipment, it is considered a valuable investigation to perform on patients visiting dental clinics. It is specifically useful in elderly patients, in whom it is desirable to evaluate swallowing function.

This study has an advantage in that it was based on data from a large number of elderly people from a wide area. However, since the participation rate of the target population was low, it is conceivable that this affected the results. Indeed, the study participants were relatively healthy elderly people who visited dental clinics on their own to undergo dental check-ups, which may have contributed to sampling bias. Therefore, differences in oral health status and swallowing function will exist between the study participants and those who do not undergo regular dental check-ups. This study examined elderly individuals ≥75 years of age, but the relationship between oral factors and swallowing function may differ in younger individuals.

This study has some other limitations. It is impossible to identify a causal relationship between oral factors and swallowing function from a cross-sectional observational study; therefore, future longitudinal studies are warranted. The RSST, which was used to evaluate swallowing function, is a screening test and cannot strictly evaluate swallowing function. It is necessary to confirm our findings regarding the association between various oral factors and swallowing function assessed using VF. Oral examinations were performed according to detailed instructions, but the interexaminer reliability of the CPI and the plaque index were not confirmed by the kappa statistics among dentists due to the extensive research area. As the evaluation of dry mouth was based on a visual assessment, directly measuring saliva secretion would strengthen our findings of an association between dry mouth and decreased swallowing function. As this study used data from dental examinations, information concerning general health status, such as ADLs, is poor. Further studies that take into consideration systemic health are required, as deterioration of swallowing function is affected by general health status in addition to oral factors.

## Conclusion

In conclusion, the present study demonstrated that oral factors such as number of teeth, denture use, dry mouth, and oral hygiene status were related to swallowing function in elderly individuals. To prevent deterioration of swallowing function, it is important to preserve as many teeth as possible.
